# Single-Center Retrospective Case Series of 33 Patients with Ophthalmic–Cerebral Embolic Stroke Following Cosmetic Facial Injection, 2017–2025

**DOI:** 10.3390/healthcare14152284

**Published:** 2026-07-27

**Authors:** Na Zheng, Yueqi Guo, Yunxia Wang, Guoen Yao, Miao Li

**Affiliations:** Department of Neurology, Fourth Medical Center of PLA General Hospital, Beijing 100048, China; zn901213@163.com (N.Z.); guoyueqi2013@163.com (Y.G.); yun0601051@163.com (Y.W.)

**Keywords:** cosmetic injection, hyaluronic acid, autologous fat grafting, cerebral infarction, ophthalmic artery

## Abstract

**Highlights:**

**What are the main findings?**

- In 33 patients with ophthalmic–cerebral embolic stroke after cosmetic facial injection, cerebral infarction predominantly involved the anterior circulation (97.0%), frequently with a multifocal/multi-territorial distribution (72.7%).
- The median onset-to-intervention time was 25.4 h, with only 7.1% of patients treated within 6 h; at discharge, ocular muscle (65.4%) and skin (63.0%) abnormalities improved more than severe visual impairment (20.7%). Critically, within our cohort, earlier intervention was not associated with visual improvement (≤24 h 3/10 vs. >24 h 2/14; Fisher *p* = 0.61; median time to intervention was 24.0 h in patients who improved vs. 26.6 h in those who did not), so activation of the pathway should not be presented as a means of restoring vision.

**What are the implications of the main findings?**

- Acute blindness after cosmetic injection, accompanied by skin ischemia and/or neurological symptoms, should trigger simultaneous activation of the stroke green channel and emergency ophthalmology.
- Given the retina’s narrow ischemic window, preventive strategies and rapid referral pathways from esthetic facilities to stroke centers are needed.

**Abstract:**

**Background/Objectives:** We aimed to describe clinical phenotypes, imaging distribution, treatment time window, and short-term outcomes of ophthalmic–cerebral artery embolic stroke caused by cosmetic facial injections or fillers in a tertiary neurology-referred cohort. **Methods:** This single-center retrospective observational case series included 33 consecutive eligible patients with ophthalmic artery embolism complicated by cerebral infarction or optic nerve ischemia after cosmetic facial injections. All 33 patients had cerebral infarction. Demographic, clinical, imaging, treatment-timing, and discharge-outcome data were extracted from medical records and analyzed descriptively. **Results:** Mean age was 31.1 ± 7.5 years (median 30.0, IQR 25.0–37.0); 29 (87.9%) were female. The injected materials were as follows: hyaluronic acid (24, 72.7%), autologous fat (8, 24.2%), and unknown (1, 3.0%). Anterior circulation involvement occurred in 32 patients (97.0%), posterior in 16 (48.5%) (an exploratory observation of uncertain mechanism; 15/16 co-occurred with anterior involvement), both in 15 (45.5%), multifocal/multi-territorial infarction in 24 (72.7%), and hemorrhagic transformation in six (18.2%). Optic nerve ischemia co-occurred in 25/33 patients (75.8%); no patient had isolated optic nerve ischemia without cerebral infarction. Median time from onset to intervention was 25.4 h (IQR 9.0–34.5); only two of 28 (7.1%) received intervention within 6 h. Within our cohort, earlier intervention was not associated with visual improvement (≤24 h 3/10 vs. >24 h 2/14; Fisher’s *p* = 0.61). At discharge, as a within-patient change between admission and discharge (not a controlled treatment-effect estimate), ocular motility abnormalities improved (17/26, 65.4%, *p* < 0.001), skin abnormalities improved (17/27, 63.0%, *p* < 0.001), and severe visual impairment improved (6/29, 20.7%, *p* = 0.031). **Conclusions:** Cosmetic facial injection/filler-related stroke is a specific type of iatrogenic embolic stroke caused by the dissemination of exogenous materials through facial–orbital–intracranial anastomotic pathways. Because the cohort was ascertained through a tertiary neurology department, the phenotype reflects the severe, brain-involved end of the spectrum and the distribution estimates are not generalizable to the full spectrum of filler-related vascular events. For patients presenting with acute blindness accompanied by skin ischemia, ocular muscle abnormalities, or neurological symptoms following cosmetic injections, early referral to both the stroke green channel and emergency ophthalmology for multidisciplinary evaluation is recommended, primarily to limit cutaneous necrosis, support extraocular muscle recovery, and monitor and control infarct progression and hemorrhagic transformation rather than to restore vision.

## 1. Introduction

Hyaluronic acid, autologous fat, and other facial fillers, once inadvertently entering the arterial system, can travel retrogradely through anastomotic branches between facial arteries and the ophthalmic artery into the central retinal artery and intracranial branches, leading to permanent blindness and even death [1,2,3]. Li et al. proposed a hemodynamic mechanism involving pressure gradients [4]. This iatrogenic embolic stroke affects young patients without traditional risk factors, with ocular, cerebral, and cutaneous involvement. Prior studies are mainly from ophthalmology or plastic surgery [1,5,6]. Thus, we analyzed 33 cases, described as “predominant anterior circulation involvement, often with posterior and multifocal involvement,” and evaluated short-term changes in ocular muscle, skin, and vision.

## 2. Materials and Methods

### 2.1. Study Design and Participants

This single-center retrospective observational case series was conducted at the Department of Neurology, Fourth Medical Center of PLA General Hospital, Beijing, China. Consecutive cases admitted between January 2017 and December 2025 were identified independently by two investigators from electronic medical records and confirmed by a third senior physician. Ethical approval was granted by the Ethics Committee of the Fourth Medical Center of PLA General Hospital (approval No. 2026KY081-KS001); informed consent was waived because the study used de-identified retrospective data with no additional intervention. Data extraction ended on 4 June 2026, after the last consecutive case admitted in December 2025. That approval was granted on 3 June 2026 and was therefore obtained retrospectively, that is, after the close of the January 2017–December 2025 admission window and before data extraction and analysis. All patients admitted in December 2025 had complete inpatient and discharge records at the time of analysis.

Patients were included if they had: (1) acute onset after facial injection or filler; (2) acute vision loss, ocular pain, ophthalmoplegia, or ocular arterial occlusion signs; (3) clinical or imaging evidence of central retinal or ophthalmic artery embolism; (4) cranial imaging showing cerebral infarction or optic nerve ischemia; and (5) key variables available. A total of 33 eligible patients were identified from the hospital case system and all were included in the final analysis, with no exclusions. All 33 included patients had cerebral infarction on cranial imaging; there were no cases of isolated optic nerve ischemia without cerebral infarction, so the case definition “ophthalmic–cerebral embolic stroke” applies to the whole cohort.

This report follows the Strengthening the Reporting of Observational Studies in Epidemiology (STROBE) statement (Supplemental Checklist in Supplementary Materials). The study size was determined by the number of consecutive eligible cases during the predefined study period; no formal sample size calculation was performed because this was a rare-event descriptive case series.

### 2.2. Variable Definitions

Data were extracted from electronic medical records and imaging or angiographic records, including age, sex, injected material, onset time, treatment timing and medications, affected vessels, infarction location, neurological symptoms, and short-term outcomes. Materials were classified as hyaluronic acid, autologous fat, or unknown.

Severe visual impairment was considered as follows: no light perception, blindness, or inability to see. Ocular muscle abnormalities included ptosis, ophthalmoplegia, and restricted movement. Skin abnormalities included livedo reticularis, cyanosis, pallor, ulceration, and necrosis. Focal deficits included documented limb weakness, numbness, aphasia, or hemiplegia. Improvement of an outcome was defined operationally as the loss, at discharge, of a categorical abnormality that was present at admission (for vision, no longer meeting the severe-visual-impairment criterion), assessed only among patients abnormal at admission (at-risk denominator).

NIHSS/mRS scores were retrospectively estimated from records; a score of 0 was assigned only when records explicitly indicated normal findings. These estimates carry a risk of underestimation and are used only for descriptive and sensitivity analyses, not as primary outcomes. Because the NIHSS captures visual loss only through the binocular visual-field item, it does not reflect monocular blindness—the defining deficit in this cohort—and therefore underestimates severity here; scores are reported for descriptive purposes only.

Infarction territories were classified by imaging: occipital lobe = posterior circulation; frontal/parietal lobe, periventricular, and basal ganglia = anterior circulation. For dual-supply lesions (e.g., posterior temporal), two neurologists determined the territory case-by-case, and a third resolved disagreements. Multifocal/multi-territorial infarction was defined as ≥2 regions/territories. Hemorrhagic transformation was defined as post-infarction hemorrhage, hemorrhagic transformation, or subarachnoid hemorrhage on imaging. Imaging analysis covered ischemic patterns, ophthalmic–cerebrovascular involvement, and hemorrhagic complications. All variables were independently assessed by two neurologists, and a third resolved disagreements.

The availability of each variable was recorded from the source records. Denominators were reported when data were incomplete (angiographic records, *n* = 27; onset-to-intervention time, *n* = 28). No missing values were imputed in the primary descriptive analyses; conservative imputation was used only in the sensitivity analysis for retrospectively estimated NIHSS and mRS scores.

To limit selection and classification bias, consecutive cases were independently identified by two investigators, imaging and clinical variables were independently assessed by two neurologists, and disagreements were adjudicated by a third reviewer. Because treatments were not standardized and subgroup sizes were small, treatment and material-group analyses were considered exploratory.

### 2.3. Statistical Methods

Continuous variables are presented as mean ± SD, median, and IQR, and categorical variables as counts and percentages. Excluding the unknown-material case, Fisher’s exact test was used for material-group comparisons and the exact McNemar test for paired pre-/post-treatment changes. A two-sided *p* < 0.05 was considered significant. Given the small sample, subgroup analyses were exploratory, with no multiple-comparison correction. Wilson 95% confidence intervals are reported for key proportions. Analyses were performed with R version 4.6.0 (R Foundation for Statistical Computing, Vienna, Austria). No multiple-comparison correction was applied, and this is reflected in the Results and Discussion as a limitation.

## 3. Results

### 3.1. Baseline and Clinical Phenotype

Thirty-three patients met the eligibility criteria during the study period and were included in the analysis (Table 1). Their mean age was 31.1 ± 7.5 years (median 30.0, IQR 25.0–37.0), and 29 (87.9%) were female. Injection materials were hyaluronic acid in 24 patients (72.7%), autologous fat in eight (24.2%), and unknown in one (3.0%). At admission, severe visual impairment was present in 29 patients (87.9%). Ophthalmic artery involvement included the main trunk in one patient, the central retinal artery in 22, and mixed involvement in four. Neurological symptoms included headache, dizziness, nausea, or vomiting in 27 patients (81.8%) and focal deficits in five patients (15.2%). Ocular muscle abnormalities occurred in 26 patients (78.8%), and skin abnormalities in 27 (81.8%). All 33 patients had cerebral infarction. Twenty-five patients (75.8%) also had optic nerve ischemia and eight had cerebral infarction without documented optic nerve ischemia; no patient had isolated optic nerve ischemia. Key proportions (Wilson 95% CI): female—87.9% (72.7–95.2); severe visual impairment at admission—87.9% (72.7–95.2); ocular muscle abnormalities—78.8% (62.2–89.3); and skin abnormalities—81.8% (65.6–91.4).

### 3.2. Imaging and Angiographic Features

**Figure 1:**
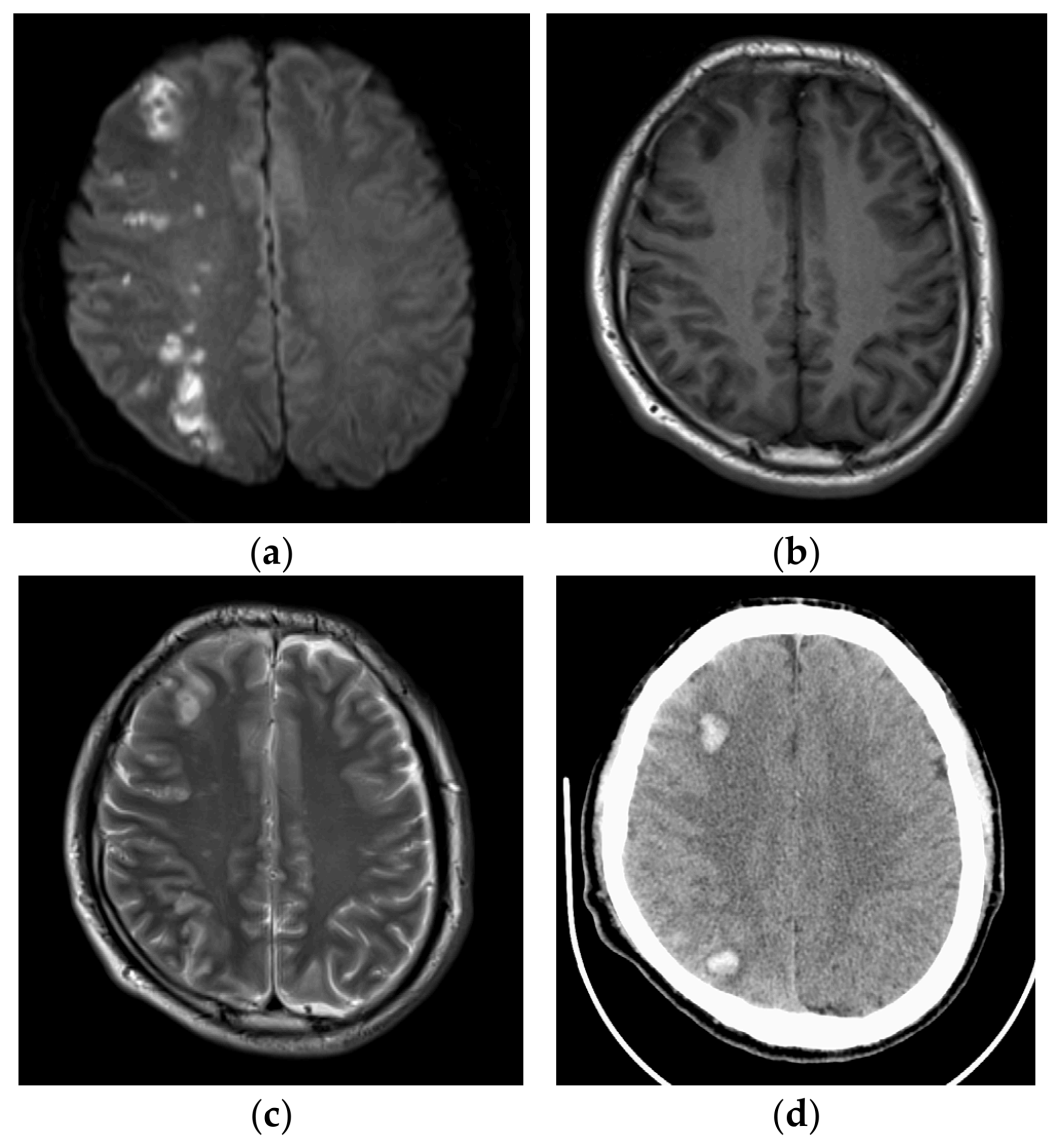
Imaging findings of cerebral infarction with hemorrhagic transformation after hyaluronic acid injection: (**a**) DWI shows multiple acute infarcts in the right frontal–parietal lobe; (**b**) T1-weighted image shows slightly prolonged T1 signal in the right frontal–parietal lobe; (**c**) T2-weighted image shows slightly prolonged T2 signal in the right frontal–parietal lobe; (**d**) CT shows hemorrhage in the right frontal–parietal lobe.

**Figure 2:**
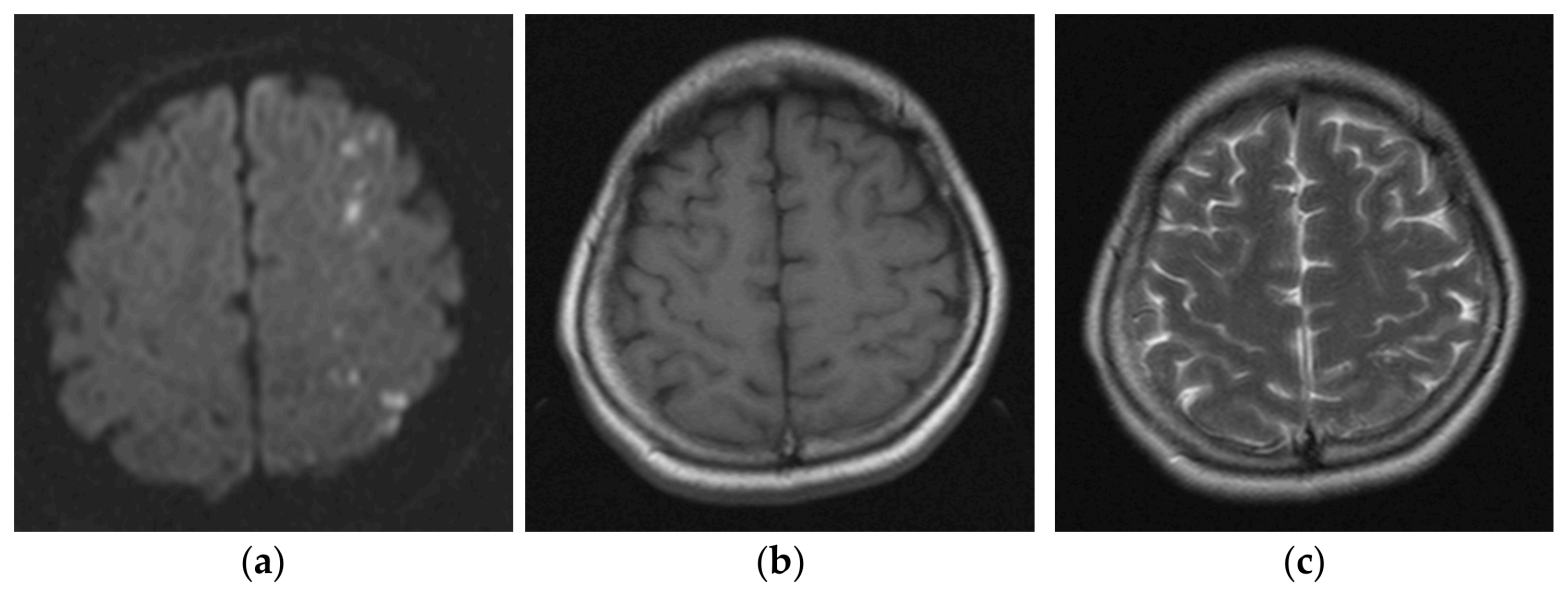
Imaging findings of cerebral infarction after fat injection: (**a**) DWI shows multiple scattered acute infarcts in the left frontal–parietal lobe; (**b**) T1-weighted image shows slightly prolonged T1 signal in the left frontal–parietal lobe; (**c**) T2-weighted image shows slightly prolonged T2 signal in the left frontal–parietal lobe.

**Figure 3:**
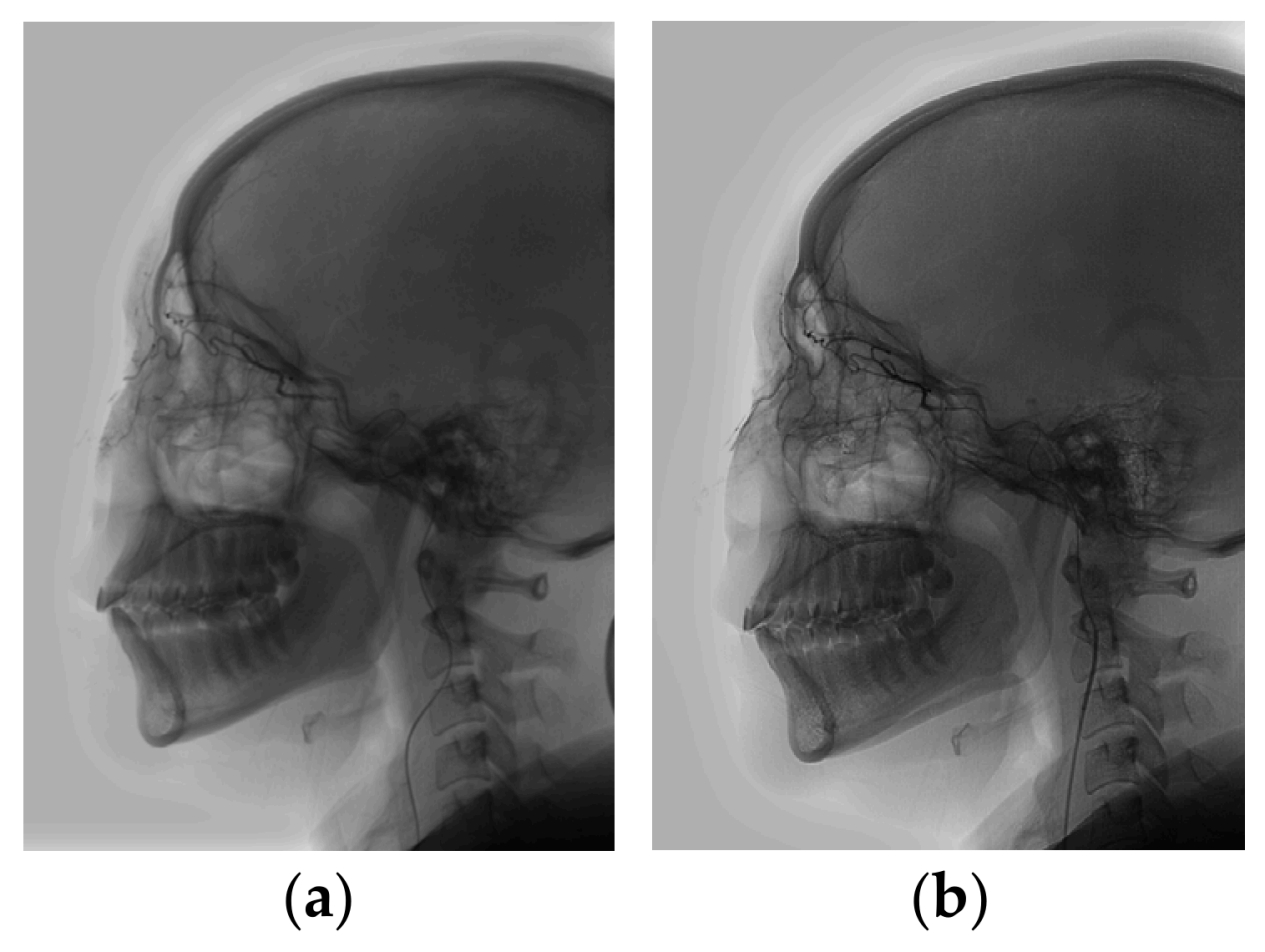
Imaging findings of cerebral infarction after hyaluronic acid injection: (**a**) pre-treatment, left internal carotid artery angiography shows a filling defect in the central retinal artery, a branch of the left ophthalmic artery; (**b**) post-treatment, left internal carotid artery angiography shows restoration of blood supply to the central retinal artery.

Anterior circulation involvement was seen in 32 patients (97.0%), posterior circulation in 16 (48.5%), and both in 15 (45.5%). Multifocal/multi-territorial infarction was seen in 24 patients (72.7%), bilateral involvement in four (12.1%), optic nerve ischemia in 25 (75.8%), and hemorrhagic transformation in six (18.2%). Three lesion patterns were observed: frontoparietal/basal ganglia; frontoparietal plus temporal–occipital; and bilateral punctate DWI hyperintensities (microembolic). DSA showed an attenuated or occluded ophthalmic trunk, a non-visualized central retinal artery, and poor external carotid perfusion. Post-intervention, ophthalmic trunk flow improved in most patients, whereas complete restoration of central retinal artery supply was achieved in only a minority. Among the 27 patients with angiographic records, central retinal artery embolism (Group B) was the most common subtype (22/27, 81.5%), presenting with acute severe visual impairment plus anterior circulation infarction in most cases. Group A (main trunk, *n* = 1) and the mixed group (*n* = 4) were too small for stable conclusions. The angiographic subgroups are defined as follows: Group A = ophthalmic artery main trunk (*n* = 1); Group B = central retinal artery (*n* = 22, 81.5% of 27); and mixed/other ophthalmic artery branches (*n* = 4). Angiographic records were available in 27/33 patients. Posterior circulation involvement (16/33, 48.5%) is reported as an exploratory observation of uncertain mechanism, because classification used a lobe-based rule without an independent imaging core laboratory and 15/16 posterior cases co-occurred with anterior involvement. Post-intervention recanalization outcomes were as follows: DSA of the ophthalmic arterial system was performed in 27/33 patients (81.8%, 95% CI 65.6–91.4); five were not catheterized, and one further patient underwent DSA of the external carotid, facial and nasopharyngeal arteries without demonstrable ophthalmic artery involvement, so that 28 patients in total were treated endovascularly. Among the 27 with ophthalmic angiograms, the occlusion target was the ophthalmic artery main trunk in one patient (Group A; 3.7%, 95% CI 0.7–18.3), central retinal artery non-visualization in 22 patients (Group B; 81.5%, 95% CI 63.3–91.8), and mixed or other ophthalmic-artery branches in four patients (Group C; 14.8%, 95% CI 5.9–32.5). After intervention, angiographic restoration of the target vessel was complete (central retinal artery re-opacified) in seven patients (25.9%, 95% CI 13.2–44.7) and partial (ophthalmic trunk and distal flow improved, with the central retinal artery not opacified or only faintly opacified) in 20 (74.1%, 95% CI 55.3–86.8); no patient failed to show any angiographic improvement (0/27, 95% CI 0.0–12.5). Cross-tabulated with visual status at discharge among the 24 angiographically studied patients who presented with severe visual impairment, improvement by discharge occurred in 1/6 patients (16.7%) after complete restoration versus 4/18 (22.2%) after partial restoration (Fisher’s exact *p* = 1.000). Angiographic restoration of the central retinal artery was therefore not associated with visual recovery in this cohort; these subgroup counts are small, and the comparison is exploratory, descriptive and non-causal (Table 2, Figure 1, Figure 2 and Figure 3).

### 3.3. Time Window, Treatment, and Material Comparisons

The onset-to-intervention time was calculable in 28 cases, with a median of 25.4 h (IQR 9.0–34.5); intervention occurred within 6 h in two patients (7.1%) and within 24 h in 13 (46.4%). Medications included local hyaluronidase in 17 patients (51.5%), interventional hyaluronidase in 22 (66.7%), papaverine in 28 (84.8%), and urokinase in six (18.2%). Among the six hemorrhagic transformations, one had received urokinase. No significant differences were found between the hyaluronic acid (*n* = 24) and autologous fat (*n* = 8) groups in this small exploratory analysis (severe visual impairment at admission was 87.5% in both groups; at discharge 66.7% vs. 75.0%, *p* = 1.000). Each therapy is reported over the full cohort with denominators: local hyaluronidase 17/33 (51.5%), intra-arterial (intervention-related) hyaluronidase 22/33 (66.7%), papaverine 28/33 (84.8%), and urokinase 6/33 (18.2%). “Intervention” is defined throughout as the first endovascular (intra-arterial) procedure performed after admission—that is, the first DSA-guided aspiration and injection session—and not first medical contact, first ophthalmological consultation, or local hyaluronidase given before transfer; onset-to-intervention time was accordingly calculable in the 28 patients treated endovascularly. Local hyaluronidase (17/33, 51.5%) was given before or on transfer, almost always at the treating cosmetic facility, as multi-point subcutaneous or intradermal infiltration of the injection site and the surrounding ischemic skin; one patient additionally received 1500 IU intravenously and one received a retrobulbar injection at another hospital. It was administered within hours of onset in most cases, and 3 days after onset in one patient. A numeric dose was retrievable in 6/17 patients (range 400–7500 IU) and was semi-quantitative in one further patient (“2.5 vials”); in the remaining 10, the brand and/or dose were not recorded. Endovascular treatment (28/33, 84.8%) was performed with a coaxial microcatheter superselectively positioned in the proximal ophthalmic artery—or, in the one patient without ophthalmic involvement, in the facial and nasopharyngeal arteries—using repeated aspiration followed by fractionated intra-arterial injection, with repeat angiography to assess flow. The sequence within each session was aspiration, then papaverine, and then hyaluronidase and/or urokinase. Intra-arterial papaverine was given to all 28 treated patients (28/33, 84.8%) as a vasodilator adjunct, at 30 mg in 25 patients, 60 mg in one, 45 mg in one, and an unrecorded dose in one. Intra-arterial hyaluronidase (22/33, 66.7%) was given for presumed hyaluronic acid embolus: among the 28 treated patients, it was administered to 20/20 with hyaluronic acid and to the single patient with unknown material, but to only 1/7 with autologous fat, for which the agent has no substrate. The dose was 1500 IU in most patients (range 500–3000 IU; 750 IU per side in the one bilateral case; unrecorded in one). Intra-arterial urokinase (6/33, 18.2%) was reserved for suspected non-hyaluronic-acid or thrombotic occlusion and was used mainly in fat embolism (4/7 treated patients with fat versus 2/20 with hyaluronic acid), at 50,000–250,000 IU. Five patients underwent a second endovascular session 2–6 days later for persistent occlusion.

Among patients with severe visual impairment at admission and a calculable onset-to-intervention time (*n* = 24), earlier intervention was not associated with visual improvement by discharge: ≤6 h 0/1; ≤24 h 3/10 (30.0%) versus >24 h 2/14 (14.3%), Fisher’s exact *p* = 0.61 (≤6 h vs. >6 h, *p* = 1.00). The median onset-to-intervention time was similar in patients who improved (24.0 h) and those who did not (26.6 h). No time threshold predicted visual recovery (Table 3).

### 3.4. Paired Outcomes and Severity Scores

Severe visual impairment improved in 6/29 patients (20.7%, *p* = 0.031). Ocular muscle abnormalities improved in 17/26 patients (65.4%, *p* < 0.001), and skin abnormalities improved in 17/27 (63.0%, *p* < 0.001). The estimated median admission NIHSS score was 2 (IQR 2–3), and the median discharge mRS score was two (IQR 0–2). In the five patients with focal deficits, the median NIHSS was three (IQR 2–5). Sensitivity analysis (conservative imputation) increased the median NIHSS from two to three and the mRS from two to three, suggesting an underestimation bias inherent to retrospective estimation. Reported with at-risk denominators and a Wilson 95% CI, severe visual impairment was identified in 6/29 patients (20.7%, 9.8–38.4); ocular muscle abnormalities in 17/26 (65.4%, 46.2–80.6); and skin abnormalities in 17/27 (63.0%, 44.2–78.5). These represent within-patient changes between admission and discharge, not a treatment-effect estimate.

Retrospectively estimated severity scores were used (separate from the primary descriptive outcomes above; not used as primary outcomes). The estimated median admission NIHSS was two (IQR 2–3) and the median discharge mRS was two (IQR 0–2); in the five patients with focal deficits, the median NIHSS was three (IQR 2–5). The conservative-imputation sensitivity analysis increased the median NIHSS from two to three and the mRS from two to three, indicating a meaningful underestimation risk. As noted, the NIHSS does not reflect monocular visual loss and understates severity in this population; a best-corrected visual acuity (logMAR) or ordinal (NLP/LP/HM/CF) scale would be preferable, but best-corrected visual acuity was not measured or recorded in a standardized manner in this retrospective cohort, and neither logMAR values nor a complete ordinal NLP/LP/HM/CF grading could be reconstructed for all patients from the source records. Visual status was documented in free-text notes of varying granularity (e.g., “no light perception”, “light perception uncertain”, “counting fingers”, or decimal acuities such as 0.04–0.8 in a minority of patients), and functional visual categories at discharge were likewise not uniformly classified. We therefore report the prespecified binary outcome (severe visual impairment: no light perception, blindness, or inability to see) and state explicitly that standardized logMAR or ordinal acuity grading was not consistently recorded and could not be derived post hoc. This is a limitation of the source dataset rather than of the analysis, and prospective registries should capture BCVA (logMAR) and NLP/LP/HM/CF at presentation and at discharge (Table 4).

## 4. Discussion

### 4.1. Recognition of a Distinct Iatrogenic Stroke Subtype

The patients in this cohort were young, predominantly female, and lacked traditional vascular risk factors. They all had a documented history of cosmetic procedures prior to symptom onset and rapidly developed a constellation of vision loss, ophthalmoplegia, skin ischemia, and cerebral infarction within a short timeframe. This profile is markedly different from atherosclerotic or cardioembolic stroke and aligns more closely with the TOAST classification of “stroke of other determined etiology,” specifically iatrogenic exogenous embolic stroke. The management of this condition requires simultaneous attention to the extremely brief ischemic tolerance window of the ophthalmic–retinal circulation, the risk of embolism across multiple intracranial vascular territories, and the local outcomes of facial skin and extraocular muscle ischemia.

### 4.2. The “Material–Vessel–Microcirculation” Composite Injury Mechanism and the Ophthalmic Artery as an Anatomical “Crossroads”

The classic mechanism is “high-pressure injection—retrograde intravascular entry—antegrade redistribution”: the filler material retrogradely enters the arterial system via anastomotic branches between the facial artery and the ophthalmic artery, and is subsequently redistributed antegradely into the central retinal artery and intracranial branches [7,8,9,10]. The hemodynamic hypothesis proposed by Li et al. elucidates that a transient imbalance between injection pressure and local arterial pressure is the key to retrograde embolism [4]. A case report by Qian et al. documented that autologous fat facial injections can lead to massive cerebral infarction [11].

Building on this, the ophthalmic artery, serving as a critical nexus between the internal carotid artery and the facial vascular anastomotic network, helps explain the clinical phenotype of simultaneous involvement of vision, ocular muscles, skin, and the brain observed in this cohort. After arising from the internal carotid artery, the ophthalmic artery sends the central retinal artery medially (supplying the retina; embolism → severe visual impairment), sends muscular branches laterally (supplying the extraocular muscles; embolism → ophthalmoplegia/ocular motility disorders), and forms anastomoses with external carotid artery facial branches via the supraorbital, supratrochlear, and dorsal nasal branches (embolism or hypoperfusion → livedo reticularis/skin necrosis). When filler material enters the ophthalmic artery or internal carotid artery system retrogradely via these anastomotic branches, it can then be distributed antegradely to ophthalmic artery branches and intracranial anterior circulation territories. Therefore, visual impairment, ophthalmoplegia, skin ischemia, and anterior circulation cerebral infarction are fundamentally the result of multidirectional dissemination of the same exogenous embolus within the ophthalmic artery and its anastomotic network.

The coexistence of multifocal infarction, livedo reticularis, ophthalmoplegia, and hemorrhagic transformation in this cohort suggests that the pathological process involves mechanical occlusion, secondary thrombosis due to endothelial injury, vasospasm, and ischemia–reperfusion injury. Hyaluronic acid may aggravate microvascular compression through water absorption and swelling, while autologous fat, with its larger particle size and insolubility, is more prone to forming high-burden proximal emboli [5,12,13]. A systematic review by Chaghamirzayi et al. further described the multisystem manifestations of fat embolism following fat grafting [14]. Therefore, this condition should be regarded as a composite ischemic injury involving the interplay of “material, vessel, and microcirculation.” This also explains the phenomenon of improved visualization of the main ophthalmic artery trunk post-intervention without necessarily restoring vision: the retina is exquisitely sensitive to ischemia, and even if proximal blood flow is restored, distal microcirculatory occlusion may have already exceeded the window of neuronal reversibility [5,8,15].

The literature suggests noteworthy differences between the emboli caused by these two materials: fat emboli are larger and more likely to become lodged in the proximal main ophthalmic artery trunk; hyaluronic acid is enzymatically degradable; and fat emboli may lead to more extensive or more rapidly progressive skin necrosis [5,12]. In this cohort, the proportions of severe visual impairment at admission were identical between the autologous fat group and the hyaluronic acid group (87.5% vs. 87.5%), and material subgroup analysis did not identify statistically significant differences. However, given the limited sample size, these data cannot verify true differences between the two materials, nor should they be interpreted as indicating equivalent clinical risk. Obtaining information on the injected material in the emergency setting remains directly significant for formulating treatment strategies.

### 4.3. Imaging Value of Multi-Pathway Dissemination

In this cohort, anterior circulation involvement was observed in 32 cases (97.0%), posterior circulation involvement in 16 cases (48.5%), and simultaneous anterior and posterior circulation involvement in 15 cases (45.5%). This distribution pattern is consistent with the anatomical relationship in which the ophthalmic artery originates from the internal carotid artery and shares the internal carotid artery system with the anterior circulation. Once exogenous emboli enter the ophthalmic artery or the lumen of the internal carotid artery via the facial–ophthalmic anastomotic network, they can subsequently be distributed with blood flow to the territories of the anterior cerebral artery and middle cerebral artery, thus accounting for the highest proportion of anterior circulation involvement.

There is no direct anatomical anastomosis between the posterior circulation (vertebrobasilar system) and the ophthalmic artery. The mechanism of posterior circulation involvement remains unclear and may be related to multidirectional dissemination from the internal carotid artery system via communicating branches or complex anastomotic networks, or may be influenced by the classification of lesions in the temporal lobe and mixed-supply territories. In this cohort, the proportion of posterior circulation involvement was lower than that of anterior circulation involvement, and most cases with posterior circulation involvement also had concomitant anterior circulation involvement, suggesting that it may not result from an independent, direct pathway. However, this study lacks case-by-case hemodynamic evidence and complete vascular pathway documentation, and further imaging verification is required. We therefore treat posterior circulation involvement as an exploratory observation of uncertain mechanism throughout and note that misclassification of dual-supply territories and fetal-type posterior cerebral artery variants cannot be excluded in the absence of an independent imaging core laboratory. Circle-of-Willis configuration was not systematically assessed in this cohort: dedicated intracranial vascular imaging (CTA/MRA or complete multi-vessel cerebral DSA runs) was not performed uniformly, and the available radiology and procedure reports did not document circle-of-Willis anatomy or fetal-type posterior cerebral artery variants in a standardized, retrievable manner. No case-level record of circle-of-Willis variation could therefore be extracted, and we cannot quantify how often a fetal-type posterior cerebral artery might account for occipital lesions classified here as posterior circulation. We report this as a limitation; it reinforces our decision to treat posterior circulation involvement as an exploratory observation of uncertain mechanism, and systematic recording of circle-of-Willis anatomy should be a requirement of future prospective series.

In this cohort, multifocal/multi-territorial infarction was present in 24 cases (72.7%), with four cases manifesting as bilateral scattered punctate DWI hyperintensities, which overlapped with the cerebral findings of fat embolism syndrome but had no predisposing factors such as long bone fractures. Therefore, in young women presenting with multi-territorial infarction and acute blindness, a history of cosmetic injections should be incorporated into the stroke etiological workup. Furthermore, the six cases of hemorrhagic transformation suggest that SWI/CT screening should be completed prior to intra-arterial therapy. Among patients with hemorrhagic transformation in this cohort, the rate of urokinase use was low (1/6). Due to the small sample size, causality cannot be determined, but vigilance is warranted regarding the potential for vascular wall injury caused by exogenous materials to increase the risk of post-thrombolysis hemorrhage.

### 4.4. Clinical Implications: Emergency Identification and Outcomes

In this cohort, 81.8% had skin ischemia, 78.8% had ocular muscle abnormalities, and 15.2% had focal neurological deficits, reflecting the same vascular event in different tissues [16]. We recommend “acute blindness after cosmetic injection + skin ischemia ± neurological symptoms” as an emergency identification anchor to trigger both the stroke green channel and the emergency ophthalmology pathway [17,18,19]. Because vision proved largely irreversible, we reframe the bedside goal as a concrete, occlusion-level- and tissue-stratified scheme: (i) main trunk/central retinal artery/mixed occlusion should be distinguished on presentation; (ii) for each target tissue, a realistic window applies—minutes for the retina (largely pre-hospital/on-site, e.g., immediate injection cessation and on-site hyaluronidase for HA), hours for extraocular muscle and skin, and the sub-acute period for monitoring infarct progression and hemorrhagic transformation; and (iii) the emphasis lies upstream, in prevention and immediate on-site response, given the retina’s very narrow ischemic window.

The median onset-to-intervention time was 25.4 h, with only 7.1% within 6 h and 46.4% within 24 h. The retina’s ischemic tolerance is far shorter than that of the cerebral penumbra, reflecting a lack of mature transfer pathways from esthetic facilities to stroke centers. Consensus statements and systematic reviews recommend immediate injection cessation, emergency ophthalmology/stroke evaluation, and material- and occlusion-level-dependent interventions (e.g., local hyaluronidase), though evidence levels remain limited [17,18,19,20]. Upstream measures should include adverse event checklists for esthetic practitioners, designated transfer hospitals, and inclusion of acute visual loss as a green channel activation criterion.

Short-term outcomes showed improvement rates of 65.4% for ocular muscle abnormalities, 63.0% for skin ischemia, and 20.7% for severe visual impairment (*p* = 0.031). Six patients no longer met the severe visual impairment criteria at discharge; occlusion site, distal microcirculatory status, and ischemia duration may influence visual outcomes, but no single determinant could be identified. Although visual improvement reached statistical significance, the degree was limited, and most patients still had severe residual impairment. Existing evidence does not demonstrate any treatment that stably reverses filler-related blindness [8,21]. Thus, clinical goals should not be limited to “recovering vision” but should also include clarifying the occlusion level, improving perfusion, reducing skin/ocular muscle ischemia, and controlling infarction progression and hemorrhage risk.

Among the six hemorrhagic transformation cases, only one had received urokinase, suggesting that hemorrhagic transformation may relate more to ischemia–reperfusion and direct vascular wall injury than to thrombolysis alone. Exogenous materials may render vessel walls more fragile; therefore, SWI/CT screening should be completed before thrombolysis, and material type and occlusion level should be incorporated into hemorrhage risk assessment.

Effective strategies should shift upstream to prevention: standardizing injection qualifications, avoiding high-risk regions, reducing high-pressure injections, and establishing hyaluronidase emergency protocols. Stroke centers should establish registries for cosmetic-injection-related strokes. The use of minimally invasive facial rejuvenation—dermal fillers, botulinum toxin, platelet-rich plasma, and PDO threads—continues to expand with rising patient demand, underscoring the need for careful patient selection, anatomical precision, and standardized safety protocols [22,23]. Even where combined minimally invasive approaches show high short-term satisfaction and low major-complication rates, these rare but severe vascular and neuro-ophthalmic complications justify structured emergency-recognition pathways, adverse-event monitoring, and stronger clinician awareness during cosmetic facial procedures [23].

### 4.5. Limitations

This study has several limitations: a single-center retrospective design with 33 cases; no independent imaging core laboratory, potentially affecting the classification of posterior circulation and mixed-supply lesions; incomplete key variables (e.g., fundus exam, long-term follow-up, injection details); retrospective NIHSS/mRS estimation with a risk of underestimation; and reliance on medical record text, introducing information bias. The material subgroup comparison is exploratory and cannot infer equivalence. Additionally, treatment interventions were not standardized across patients, as different therapeutic strategies (e.g., local hyaluronidase, intra-arterial hyaluronidase, papaverine, and urokinase) were administered at the discretion of the treating physicians, precluding meaningful comparison of treatment efficacy. The absence of a control group further limits the ability to draw causal inferences regarding treatment effects. Finally, long-term follow-up data beyond discharge were unavailable, and the short-term outcomes reported here may not reflect the ultimate functional recovery of patients. Future multicenter prospective registries with standardized data collection and independent imaging review are needed. As a single-center tertiary referral cohort, the study may overrepresent severe presentations; its findings may not be generalizable to less severe events or other care settings. Because the cohort was ascertained solely through a tertiary neurology department, only patients whose emboli reached the brain were captured, and isolated ocular cases are structurally absent; the distribution estimates (e.g., multifocal infarction 72.7%) are likely biased upward and are not generalizable to the full spectrum of filler-related vascular events. The material comparison is exploratory and, with only eight autologous fat patients and uniformly non-significant *p*-values, cannot establish equivalence. As treatments were non-standardized with no control group and no long-term follow-up, no causal treatment inference is possible.

## 5. Conclusions

Cosmetic-injection-related ophthalmic–cerebral embolic stroke is a rare but disabling iatrogenic event involving ocular, cerebral, and cutaneous damage. Cerebral infarction predominantly affects the anterior circulation, often with a multifocal/multi-territorial distribution; posterior circulation involvement requires further confirmation. After treatment, ocular muscle and skin abnormalities show greater improvement than visual impairment. For acute blindness accompanied by skin ischemia, ocular muscle abnormalities, or neurological symptoms, early activation of the stroke green channel and a joint emergency ophthalmology assessment are recommended. These estimates apply to a neurology-referred population and are not generalizable to the full spectrum; posterior circulation involvement remains an exploratory observation of uncertain mechanism; and the discharge changes represent within-patient course, not demonstrated treatment efficacy. Given that earlier intervention was not associated with visual recovery in this cohort, the pathway is recommended primarily to limit cutaneous necrosis, support extraocular muscle recovery, control infarct progression and hemorrhage—and, above all, to drive prevention—rather than to restore vision.

## Figures and Tables

**Table 1:** Baseline characteristics and clinical phenotypes at admission.

Value	Number
Age, mean ± SD (median [IQR]), years	31.1 ± 7.5 (30.0 [25.0–37.0])
Female	29 (87.9)
Injection material: Hyaluronic acid	24 (72.7)
Injection material: Autologous fat	8 (24.2)
Injection material: Unknown	1 (3.0)
Severe visual impairment at admission	29 (87.9)
Abnormal ocular muscle/eye movement	26 (78.8)
Skin abnormalities	27 (81.8)
Headache/dizziness/nausea/vomiting	27 (81.8)
Focal neurological deficits	5 (15.2)

Note: Unless otherwise specified, data are presented as counts (%).

**Table 2:** Imaging characteristics, time window, and interventional treatment.

Value	Number
Anterior circulation involvement	32 (97.0)
Posterior circulation involvement	16 (48.5)
Simultaneous anterior/posterior circulation involvement	15 (45.5)
Multifocal/multiregional infarction	24 (72.7)
Bilateral lesions	4 (12.1)
Optic nerve ischemia	25 (75.8)
Hemorrhagic changes (hemorrhagic transformation, subarachnoid hemorrhage)	6 (18.2)
Calculable time from onset to intervention	28
Time from onset to intervention (median [IQR], hours)	25.4 (9.0–34.5)
Intervention within 6 h	2/28 (7.1)
Intervention within 24 h	13/28 (46.4)
Hyaluronidase (local administration)	17 (51.5)
Papaverine	28 (84.8)
Hyaluronidase (intervention-related administration)	22 (66.7)
Urokinase	6 (18.2)

Note: IQR denotes the interquartile range.

**Table 3:** Exploratory comparison between the hyaluronic acid group and autologous fat group.

Variable	Hyaluronic Acid Group (*n* = 24)	Autologous Fat (*n* = 8)	*p*
Severe visual impairment at admission	21 (87.5)	7 (87.5)	1.000
Ocular muscle abnormalities at admission	19 (79.2)	6 (75.0)	1.000
Skin abnormalities at admission	21 (87.5)	5 (62.5)	0.148
Headache/dizziness/nausea/vomiting	19 (79.2)	7 (87.5)	1.000
Focal neurological deficits	3 (12.5)	2 (25.0)	0.578
Anterior circulation involvement	24 (100.0)	7 (87.5)	0.250
Posterior circulation involvement	13 (54.2)	3 (37.5)	0.685
Multifocal/multiregional infarction	20 (83.3)	4 (50.0)	0.152
Hemorrhagic changes	6 (25.0)	0 (0)	0.296
Optic nerve ischemia	19 (79.2)	5 (62.5)	0.378
Severe visual impairment at discharge	16 (66.7)	6 (75.0)	1.000

Note: Data are presented as counts (%); *p*-values were calculated using Fisher’s exact test; all analyses are exploratory and no multiple-comparison correction was performed.

**Table 4:** Paired comparisons of short-term outcomes before and after treatment.

Outcome	Admission	Discharge	Improvement, *n*/At-Risk (%)	Worsening	*p*
Severe visual impairment	29 (87.9)	23 (69.7)	6/29 (20.7)	0	0.031
Ocular muscle abnormalities	26 (78.8)	10 (30.3)	17/26 (65.4)	1	<0.001
Skin abnormalities	27 (81.8)	10 (30.3)	17/27 (63.0)	0	<0.001

Note: *p*-values were calculated using the exact McNemar test.

## Data Availability

This single-center retrospective case series (33 cases) does not share original data publicly due to patient privacy and Ethics Committee requirements. De-identified materials may be available from the corresponding author upon reasonable request, subject to ethics approval.

## Supplementary Materials

The following supporting information can be downloaded at: https://www.mdpi.com/article/10.3390/healthcare14152284/s1.

## Abbreviations

The following abbreviations are used in this manuscript:

CT	Computed tomography
DSA	Digital subtraction angiography
DWI	Diffusion-weighted imaging
HA	Hyaluronic acid
ICA	Internal carotid artery
IQR	Interquartile range
mRS	Modified Rankin Scale
NIHSS	National Institutes of Health Stroke Scale
STROBE	Strengthening the Reporting of Observational Studies in Epidemiology
SWI	Susceptibility-weighted imaging
TOAST	Trial of Org 10172 in Acute Stroke Treatment
